# Gene-network based analysis of human placental trophoblast subtypes identifies critical genes as potential targets of therapeutic drugs

**DOI:** 10.1515/jib-2023-0011

**Published:** 2023-12-22

**Authors:** Andreas Ian Lackner, Jürgen Pollheimer, Paulina Latos, Martin Knöfler, Sandra Haider

**Affiliations:** Department of Obstetrics and Gynecology, Maternal-Fetal Immunology Group, Medical University of Vienna, Vienna, Austria; Center for Anatomy and Cell Biology, Medical University of Vienna, Vienna, Austria; Department of Obstetrics and Gynecology, Reproductive Biology Unit, Medical University of Vienna, Vienna, Austria

**Keywords:** WGCNA, placental development, drug-gene interaction

## Abstract

During early pregnancy, extravillous trophoblasts (EVTs) play a crucial role in modifying the maternal uterine environment. Failures in EVT lineage formation and differentiation can lead to pregnancy complications such as preeclampsia, fetal growth restriction, and pregnancy loss. Despite recent advances, our knowledge on molecular and external factors that control and affect EVT development remains incomplete. Using trophoblast organoid *in vitro* models, we recently discovered that coordinated manipulation of the transforming growth factor beta (TGFβ) signaling is essential for EVT development. To further investigate gene networks involved in EVT function and development, we performed weighted gene co-expression network analysis (WGCNA) on our RNA-Seq data. We identified 10 modules with a median module membership of over 0.8 and sizes ranging from 1005 (M1) to 72 (M27) network genes associated with TGFβ activation status or *in vitro* culturing, the latter being indicative for yet undiscovered factors that shape the EVT phenotypes. Lastly, we hypothesized that certain therapeutic drugs might unintentionally interfere with placentation by affecting EVT-specific gene expression. We used the STRING database to map correlations and the Drug-Gene Interaction database to identify drug targets. Our comprehensive dataset of drug-gene interactions provides insights into potential risks associated with certain drugs in early gestation.

## 1 Introduction

**Figure 1:**
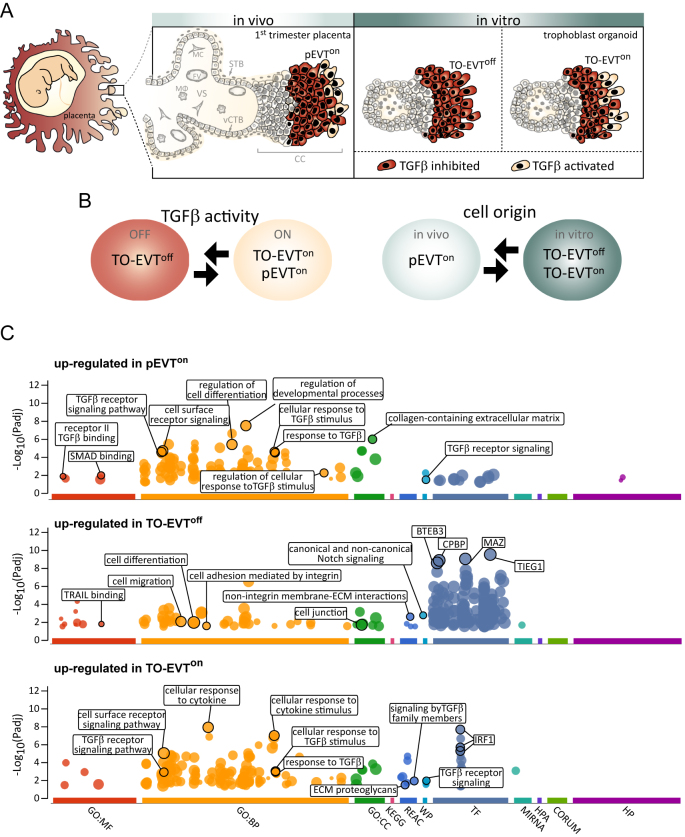
Maturation of in vitro-derived TO-EVT requires TGFβ activation. (A) Illustration of placental EVTs (pEVT) and trophoblast organoid EVTs (TO-EVT), marked in red. Villous cytotrophoblasts (vCTB) proliferate to form a cell column (CC) and differentiate into pEVT^on^. TO-EVT are differentiated under TGFβ-inhibited (TO-EVT^off^) and TGFβ-activated (TO-EVT^on^) conditions. Please note that only a subset of distally located EVTs display activated TGFβ signaling (beige). (B) EVT populations for establishing TGFβ- and cell origin-dependent WGCNA gene networks. (C) Manhattan plots depicting the g:Profiler results of enriched pathways in pEVT^on^, TO-EVT^off^, and TO-EVT^on^. Circle size correlates with the size of the enriched pathway while the *y*-axis depicts the adjusted *p*-value as a negative decadic logarithm. Pathways of interest are encircled and named. VS, villous stroma; MC, mesenchymal cell; MØ, macrophage; vCTB, villous cytotrophoblasts; STB, syncytiotrophoblast; CC, cell column; pEVT, placental extravillous trophoblast; TO-EVT, trophoblast organoid-derived EVT; GO:MF, gene ontology: molecular function; GP:BP, gene ontology: biological process; GO:CC, gene ontology: cellular component; KEGG; Kyoto encyclopedia of genes and genomes; REAC, reactome; WP, WikiPathways; TF, transcription factor; MIRNA, MicroRNA; HPA, human protein atlas; CORUM, comprehensive resource of mammalian protein complexes; HP, human phenotype.

The human placenta is a unique transiently existing organ that acts as lungs, liver, gut, kidney, and endocrine glands for the developing fetus, supplying oxygen and nutrients, and eliminating waste products [1, 2]. During early gestation, epithelial villous cytotrophoblasts give rise to a layer of multinucleated hormone-producing syncytiotrophoblasts (STBs), and migratory extravillous trophoblasts (EVTs) (Figure 1A). EVTs invade the maternal decidua, the superficial uterine mucosal layer, transforming maternal arteries into wide-lumen, low-pressure vessels to ensure a smooth and adequate supply of maternal blood [3, 4]. Additionally, EVTs reshape the maternal immune system to ensure the acceptance of the fetal semi-allograft [5]. Failures in EVT differentiation are noticed in pregnancy complications such as preeclampsia, fetal growth restriction, and early pregnancy loss exposing the mother and the baby to risk for immediate life-threatening conditions during pregnancy and severe complications later in life [6–10]. EVT differentiation is initiated by the development of so-called villous cell columns that attach to the maternal uterus (decidua) (Figure 1A). Before EVT detach from the column to penetrate the decidua, these cells undergo various stages of differentiation to form a highly invasive phenotype.

In this context, transforming growth factor beta signaling (TGFβ) was identified as one of the key regulators orchestrating EVT maturation [11, 12]. Our recent studies on trophoblast organoids (TO) demonstrated that EVTs lacking TGFβ signaling develop into hyper-invasive, spindle-shaped cells, while TGFβ-activated EVTs acquire a less-migratory, secretory phenotype, more closely mimicking the *in vivo* placental EVT (pEVT) phenotype. Sequential activation of TGFβ signalling of TO-EVT is essential to reproduce correct EVT differentiation *in vitro* (Figure 1A) [13].

Transcriptional profiling is a powerful tool for identifying physiological and pathological tissue gene signatures and their regulatory mechanisms. While the DESeq2 workflow for analysis of bulk RNA sequencing data focuses on differentially expressed genes, the Weighted Gene Co-expression Network Analysis (WGCNA, version 1.71) algorithm identifies clusters of genes with similar expression patterns and reveals related biological functions [14]. To date, WGCNA has been employed for identifying gene co-expression networks in physiological processes such as lactation [14], in various cancers to determine therapeutic targets and biomarkers [15–18], and for investigating transcriptional regulators including micro and long non-coding RNAs [19, 20]. To gain more insights into the processes orchestrating EVT maturation, we employed WGCNA with our recent RNA-Seq data, where we compared isolated EVTs from first-trimester placental tissue (pEVTs) to EVTs derived from trophoblast organoids (TO-EVTs) from the same donors [13]. Our first aim was to decipher the complex gene networks involved in TGFβ-dependent EVT maturation and identify any remaining differences between *in vivo* EVTs and *in vitro* TO-EVTs. Our second aim was to investigate external factors that could affect adequate EVT differentiation and placentation in early pregnancy. Only about 30 % of all human pregnancies result in a live birth. Besides known factors such as genetic aberration, infections, and immune rejection, about half of the patients will remain without a diagnosis [21]. A possible explanation might be external factors such as pharmaceutical drugs that unintentionally target critical EVT-specific genes in early weeks of gestation, thereby jeopardizing adequate placentation. We queried the STRING database to map gene interactions and the Drug-Gene Interaction database to identify potential drug targets. These findings provide a basis for high-throughput testing to identify possible cross-reactions of pharmaceuticals that could harm placentation in early pregnancy.

## 2 Materials and methods

### 2.1 Data collection

We performed bioinformatic analysis on RNA-Seq data sets (*n* = 16) from EVTs isolated either from first-trimester placental tissue (pEVTs) or derived from corresponding trophoblast organoids (TO), encompassing three distinct populations (Figure 1A and B) [13]: (1) *in vivo* placental extravillous trophoblasts (EVTs) comprising both TGFβ-inactive and TGFβ-active EVTs (pEVT^on^, *n* = 4) due to a TGFβ-activating *in vivo* environment, (2) *in vitro* trophoblast organoid (TO)-derived EVTs that were inhibited for TGFβ signalling (TO-EVT^off^, *n* = 8), and (3) in vitro-derived TO-derived EVTs, exposed to TGFβ-activating conditions comprising both TGFβ-inactive and TGFβ-active TO-derived EVTs (TO-EVT^on^, *n* = 4).

### 2.2 Weighted gene co-expression network analyses (WGCNA)

We used our recent RNA-Seq data sets from pEVT^on^, TO-EVT^off^, and TO-EVT^on^ [13] to run WGCNA with parameters used in Mohr et al. [22]. After creating a DESeq2 (version 1.34.0) [23] object, we filtered out genes with very low counts (<10) in over 10 % of samples and performed variance stabilizing transformation, followed by batch correction using the limma package [24]. Next, we created an eSet object with feature data described in Figure 1 and applied additional filtering using two-Gaussian filtering. To improve the filtering process, we calculated a threshold using the package mixtools (version 2.0.0) [25]. We then removed outliers, defined by an absolute *z*-normalized inter-sample connectivity (*zK*) higher than 1.96, as recommended by Oldham et al. [26].

The WGCNA parameters included a “signed” network type, β: 12, deepsplit: 2, correlation: “bicor”, and pamStage: TRUE. Modules were calculated and genes with the absolute correlation between genes and module eigengenes below 0.8 were pruned until the network stabilized. We pruned genes with an absolute correlation between genes and module eigengenes below 0.8 until the network stabilized. To verify the association of calculated modules with phenotype information, we employed a mixed-effect model and calculated *t*-tests as described by Li et al. [27]. We calculated a normalized intramodular connectivity (kWithin), which is the module connectivity divided by the maximal connectivity as shown in the following formula, with *α* as a value for the adjacency in the co-expression network:

$$\text{kWithin}\cdot {\text{norm}}_{i}=\frac{\underset{i\in \text{module}}{\sum }\alpha_{i}}{\max\left(\underset{i\in \text{module}}{\sum }\alpha_{i}\right)}$$

We identified hub genes by selecting the top 10 % of genes based on their kWithin values for each module. We then used the STRING database (version 2.4.2) to map gene interactions and the Drug-Gene Interaction database to identify potential drug targets [28–31].

## 3 Results and discussion

### 3.1 g:Profiler identified enriched biological pathways in EVT populations

To understand the differences between EVT subsets, we conducted g:Profiler analyses and created Manhattan plots to visualize functional enrichment across multiple categories, such as molecular function, biological process, cellular compartment, and human phenotype ontology (Figure 1C). To this end, we studied two stages of EVT differentiation in TO-EVTs, including TGFβ-inhibited (TO-EVT^off^) and TGFβ-activated (TO-EVT^on^), and compared those with isolated, primary pEVTs (pEVT^on^). We found that the TO-EVT^off^ subset showed enrichment of “canonical and non-canonical Notch signaling”. During EVT formation, trophoblasts undergo certain steps of differentiation orchestrated by various signaling pathways. Among these active NOTCH1 signaling was demonstrated as a prerequisite for initiating and stabilizing EVT formation [32]. Hence, the up-regulation of Notch signaling in TO-EVT^off^ might indicate an accumulation of an immature EVT phenotype. Additionally, functional enrichment analysis revealed potentially increased migratory properties in TO-EVT^off^, with pathways related to cell migration and adhesion being upregulated. These results align with our previous findings that inhibition of TGFβ prompts an invasive, less mature EVT phenotype [13]. When comparing pEVT^on^ and TO-EVT^on^, we observed a significant overlap in upregulated pathways related to TGFβ signalling, confirming that pEVT^on^ exhibit a TGFβ signature and that TO-EVT require TGFβ activation to resemble *in vivo* pEVT^on^.

### 3.2 Weighted gene co-expression network analysis (WGCNA) of EVT RNA-Seq data

**Figure 2:**
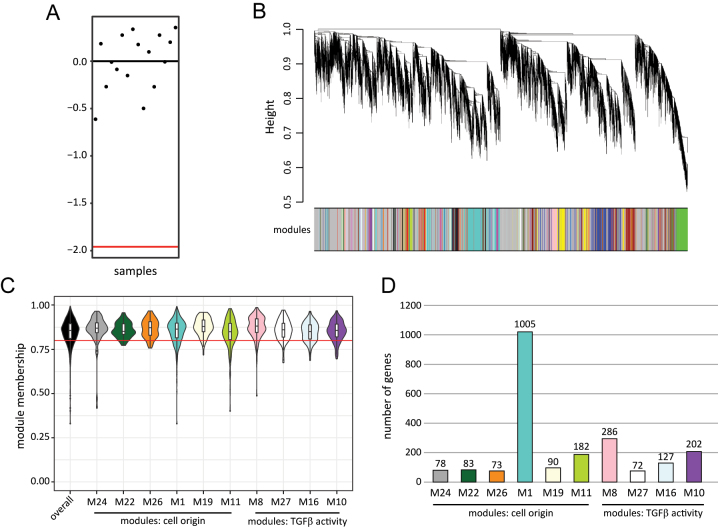
WGCNA identifies 10 modules in EVT populations. (A) *z*-normalized intersample connectivity (zKonnectivity). The red line denotes a zKonnectivity of −1.96. (B) Dendrogram showing the euclidian distance between all detected modules. Each branch depicts a gene assigned to the respective modules. Grey areas represent genes not assigned to any module. (C) Violin plots depicting the membership score of selected modules: module membership for the entire network (overall), and for the cell origin modules M24, M22, M26, M1, M19, M11, and for TGFβ modules M8, M27, M16, M10. The red line at 0.8 indicates the defined cut-off for module selection. (D) Bar graph displaying the number of genes within the modules.

To explore the relationships between different gene sets (modules), we used WGCNA to analyse the RNA-Seq data of pEVT^on^, TO-EVT^off^, and TO-EVT^on^. We also looked for significant differences between female and male samples. First, we checked that all samples had a zKonnectivity above −1.96 (Figure 2A). We then excluded genes with low expression levels across all samples, resulting in a co-expression network of 7743 genes. Using an iterative approach by Mohr et al. [26], we identified 50 modules (M) containing between 1005 (M1) and 33 (M50) genes (Figure 2B). All modules and assigned genes are listed in the Supplementary Table 1. We calculated associations between pEVT^on^ and TO-EVT^on^ from female and male donors, and the module eigengenes (Supplementary Figure 1). Surprisingly, in this study, no modules exhibited statistically significant variations between samples differentiated by fetal sex. However, potential differences in module expression relative to fetal sex may be characterized by a small effect size. Taken this into account, the sample size of the current investigation may be too small to detect such nuanced alterations. This underscores the necessity for subsequent investigations with augmented sample sizes enhancing statistical power to elucidate a potential influence of fetal sex on the modules examined herein. Hence, we did not investigate sex-specific gene expression networks further. However, we identified 10 modules (M1, M8, M10, M11, M16, M19, M22, M24, M26, and M27) that were associated with *TGFβ activity* and *cell origin*, and we selected these for further analysis (Figure 2C and D). These modules had a median module membership of above 0.8, indicating that the gene assignments were stable.

#### 3.2.1 TGFβ-dependent eigengene modules

**Figure 3:**
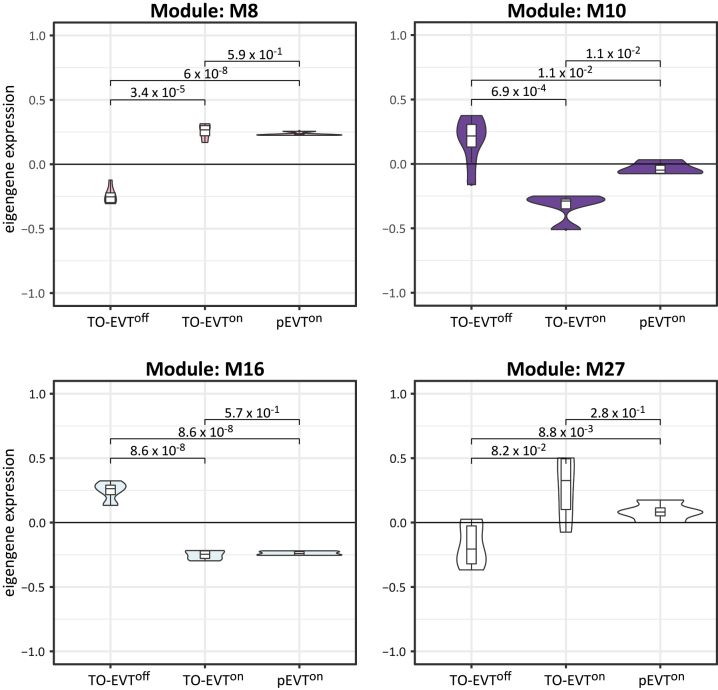
Eigengene plots of TGFβ-associated modules. Plots are colored according to the module color. *P*-values between pEVT^on^, TO-EVT^off^, and TO-EVT^on^ are depicted in the plots.

We identified four gene modules (M8, M10, M16, and M27) associated with TGFβ activity, consisting of 286, 202, 127, and 72 network genes, respectively (Figure 3, Supplementary Figure 2). Modules M8 and M27 showed increased eigengene expression in response to TGFβ signalling, while M10 and M16 exhibited reduced eigengene expression. Notably, we did not find significant differences between pEVT^on^ and TO-EVT^on^. Within the module network genes of M8 and M27, we identified several well-known TGFβ signalling-associated genes expressed in EVTs [13] including SKI, SKIL, NUAK1, FN1, HPGD, ESAM, KRT7, LITAF, ID1, and CLIC4 [33–42]. In contrast, the network genes of M10 and M16 were enriched in genes associated with an immature EVT phenotype, including PEG10, FZD6, FREM1, CDK2, CDH5, ITGA6, ITGB4, and COMT [32, 43], [44], [45], [46], [47]. These findings reasserted that *in vitro* activation of the TGFβ pathway promotes a pEVT^on^-like gene signature.

#### 3.2.2 Cell origin-dependent eigengene modules

**Figure 4:**
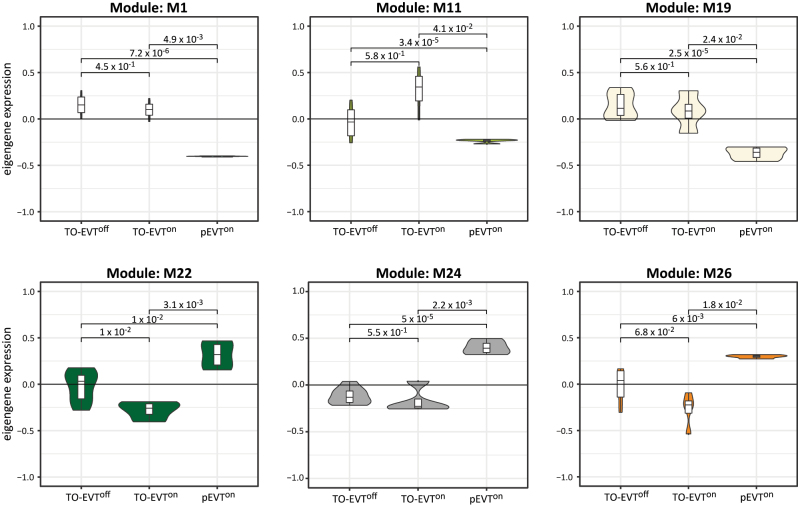
Eigengene plots of cell origin-associated modules. Plots are colored according to the module color. *P*-values between pEVT^on^, TO-EVT^off^, and TO-EVT^on^ are depicted in the plots.

Although activation of TGFβ signalling led to a significant convergence of TO-EVT^on^ genotypes to their *in vivo* counterparts, our WGCNA analyses revealed six modules that showed expressional differences between *in vivo* pEVTs and in vitro-derived TO-EVTs (Figure 4, Supplementary Figure 3). In these modules no significant differences were found between TO-EVT^off^ and TO-EVT^on^. We observed downregulated eigengene expressions in M1 (1005 network genes), M11 (182 network genes), and M19 (90 network genes) in pEVT^on^, while M22 (83 network genes), M24 (78 network genes), and M26 (73 network genes) showed increased eigengene expressions in pEVT^on^. These results suggest that the current TO-EVT culture conditions do not fully replicate pEVT^on^ maturation, and our module-based data might provide insights for identifying the complete spectrum of factors that shape pEVT^on^ genotypes in future studies.

### 3.3 Module network genes are targeted by pharmaceutical drugs

**Figure 5:**
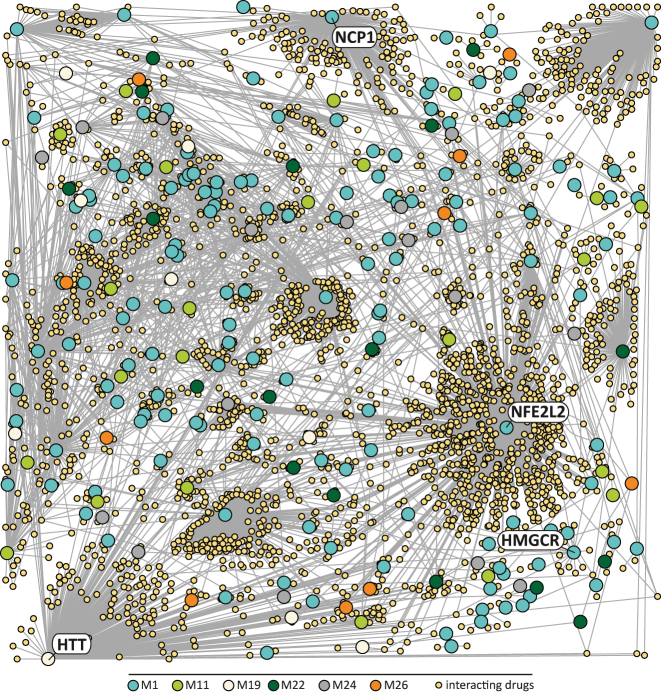
Drug-gene interaction plot for cell origin-associated modules. Network plot depicting module network genes and interacting drugs. Module network genes are colored according to the module color. *Niemann-Pick-C1* (*NCP1*), *nuclear factor erythroid 2 Like BZIP transcription factor 2* (*NFE2L2*), *3-Hydroxy-3-Methylglutrylo-CoA Red* (*HMGCR*), and *huntingtin* (*HTT*) are depicted in the interaction plot.

Our final aim was to investigate the susceptibility of EVT-specific genes to pharmaceutical drugs. We analysed the network genes of all modules (M1 – M50) using the Drug-Gene Interaction and STRING database to identify genes that can be targeted by drugs (Supplementary Table 2). Our findings revealed that numerous EVT-related genes can be affected by various drugs, such as inhibitors, modulators, agonists, inducers, antibodies, antagonists, suppressors, antisense oligonucleotides, or interacting molecules. The complexity of potential interactions between module network genes and drugs is illustrated in Figure 5 (M1, M11, M19, M22, M24, and M26) and Supplementary Figure 4 (M8, M10, M16, and M27). We further identified important drug targets from EVT network genes across the detected modules, including ITGB1 (M4, Supplementary Table 2), and HMGCR (M1, Figure 5), which have been associated with important EVT functions.

Over 800 distinct drugs have been identified to potentially interact with nuclear factor erythroid 2-like BZIP transcription factor 2 (*NFE2L2*, M1, Figure 5 and Supplementary Table 2). In human placentas, NRF2 (*NFE2L2*) has been detected in extravillous trophoblasts, with increased protein levels observed in endovascular and interstitial trophoblasts from placentas affected by fetal growth restriction and preeclampsia [48]. NRF2 plays a crucial role in the VEGF-NRF2-mediated oxidative stress response by regulating the expression of protective genes, such as heme oxygenase-1 and glutathione peroxidase [49]. As early placentation primarily occurs under hypoxic conditions, defence against reactive oxygen species becomes essential from the 9th/10th week onwards, when maternal blood begins to flow into the intervillous space [50]. In summary, drug-induced alterations in NRF2 levels may pose an unidentified risk for the basal defence mechanisms against oxidative stress during early placentation.

During pregnancy, Niemann-Pick-C1 (*NPC1*, M1, Figure 5, Supplementary Table 2) participates in the uptake of cholesterol, a vital nutrient necessary for fetal growth [51]. Although current knowledge regarding NPC1 function in placental physiology is limited, sequencing data reveal high RNA expression levels of *NPC1* in trophoblast subtypes, suggesting a role in the transport of cholesterol to the foetus during human pregnancy [13, 52]. Consequently, impaired NPC1 expression could negatively impact fetal nutrition throughout gestation.

Nearly 200 drugs potentially target huntingtin (*HTT*, M19, Figure 5, Supplementary Table 2), a gene originally identified in the context of Huntington’s disease and involved in various cellular processes such as organelle transport, spindle pole assembly during mitosis, and protein transport [53]. Gene expression profiles reveal high expression of *HTT* in trophoblast subtypes, particularly in extravillous trophoblasts [13, 52]. While the functions of HTT in human trophoblasts have not yet been demonstrated, mice lacking huntingtin exhibited impaired trophoblast giant cell differentiation [54]. These findings suggest that drug-induced alterations in HTT expression could disrupt trophoblast differentiation, which is essential for optimal maternal adaptations during pregnancy.

Integrin beta 1 (*ITGB1*, M4, Supplementary Table 2) is expressed at high levels in endovascular extravillous trophoblasts (EVTs) deeply embedded within maternal decidual tissues [55, 56]. This increased expression is believed to be necessary for the trophoblasts to withstand and migrate against maternal blood flow [56]. Our Drug-Gene Interaction database query identified eight pharmaceuticals that have the potential to target ITGB1, including antagonists (e.g., Firategrast, Volociximab, Intetumumab), inhibitors (e.g., Abituzumab, Natalizumab), and antibodies (e.g., Etaracizumab). Firategrast, for example, is a small-molecule antagonist of the integrin α4β1, which is an integrin dimer composed of ITGA4 and ITGB1 and is used to reduce trafficking of lymphocytes into the central nervous system for the treatment of multiple sclerosis [57]. Through its antagonistic effect on ITGB1, it could interfere with the establishment of the maternal-fetal interface and compromise fetal growth.

One interesting pharmacological target that has come to our attention is the interleukin-1 receptor type 1 (*IL1R1*; M8, Supplementary Figure 4, Supplementary Table 2). Evidence suggests that *IL1R1* is upregulated during trophoblast differentiation and exhibits high expression levels in extravillous trophoblasts (EVTs). Our prior laboratory experiments have demonstrated that trophoblast motility increases upon stimulation with one of its ligands, IL-1β. Additionally, we observed that IL-1β stimulation induces the expression of urokinase plasminogen activator (uPA), plasminogen activator inhibitor-1 (PAI-1), and PAI-2 in trophoblast cells. These findings suggest that the IL1R1 signalling pathway and the subsequent activation of the plasminogen system may be crucial for ensuring proper decidual invasion [58]. Anakinra, a recombinant form of IL-1Ra, directly binds to and blocks IL1R1. This compound is utilized to treat pro-inflammatory states in various diseases, including familial Mediterranean fever, rheumatoid arthritis, and cryopyrin-associated periodic syndromes. However, data regarding the use of anakinra during pregnancy are limited. Consequently, the European Medicines Agency recommends avoiding its use in pregnant patients and patients with childbearing potential who are not using contraception. In conclusion, potential effects Anakinra on EVT invasion may have significant implications for the establishment of the maternal-fetal interface [59].

We have identified 3-Hydroxy-3-Methylglutrylo-CoA Reductase (*HMGCR*, M1, Figure 5, Supplementary Table 2) as a target for 19 different drugs, primarily statins, which inhibit cholesterol synthesis. Only recently EVTs were shown to exhibit increased levels of free and esterified cholesterols, the precursors of steroid hormones such as progesterone [60]. Placental progesterone expression is considered a key factor supporting pregnancy maintenance in the first weeks of gestation [61]. Hence, reduced progesterone expression caused unintentionally by statins may pose a severe risk for successful pregnancy outcomes. Our findings align with a recent meta-analysis, which found higher rates of spontaneous abortions in patients exposed to statins [62].

## 4 Conclusions

In this study, we conducted a comprehensive bioinformatic analysis based on gene expression profiles from *in vivo* EVTs and *in vitro* TO-derived EVTs from first-trimester placental tissue. Using WGCNA, we identified expected TGFβ-dependent gene networks confirming our previous studies where we demonstrated the importance of the signalling pathway in EVT maturation [13]. However, we also detected large gene networks specific to the cells’ origin still pointing out differences between *in vivo* and in vitro-derived EVTs despite TGFβ activation. These findings indicate that while activating TGFβ signalling in TO-EVT largely resembles pEVT gene expression profiles, further studies are required to better mimic *in vivo* EVT phenotypes. Surprisingly, we found that fetal sex did not result in sex-specific gene modules. Our drug-gene interaction analysis identified several EVT-specific genes as potential drug targets, including *ITGB1, RXRA*, and *HMGCR*. Compromised placentation provoked by drug side effects might be one explanation for idiopathic early pregnancy loss. Consequently, there is an urgent need for data collection and further investigation of unintended pharmaceutical effects counteracting EVT development and differentiation. We propose using TO-EVT cultures as a highly reliable, animal-free model for testing pharmaceutical effects on EVT formation and function to protect and support early pregnancy.

## Supplementary Material

Supplementary Material Details

Supplementary Material Details

Supplementary Material Details
