# Supplementary material for: Gene-network based analysis of human placental trophoblast subtypes identifies critical genes as potential targets of therapeutic drugs
Source: J Integr Bioinform. 2023 Dec 22;20(4):20230011. doi: 10.1515/jib-2023-0011 (PMC10777358; doi:10.1515/jib-2023-0011)
Supplement: Supplementary file 1 — Supplementary Material Details [file j_jib-2023-0011_suppl_001.docx]

**Supplementary File**

**Gene-network based analysis of human placental trophoblast subtypes identifies critical genes as potential targets of therapeutic drugs**

Andreas Ian Lackner^1^, Jürgen Pollheimer^1^, Paulina Latos^2^, Martin Knöfler^3^, and Sandra Haider^3*^

**
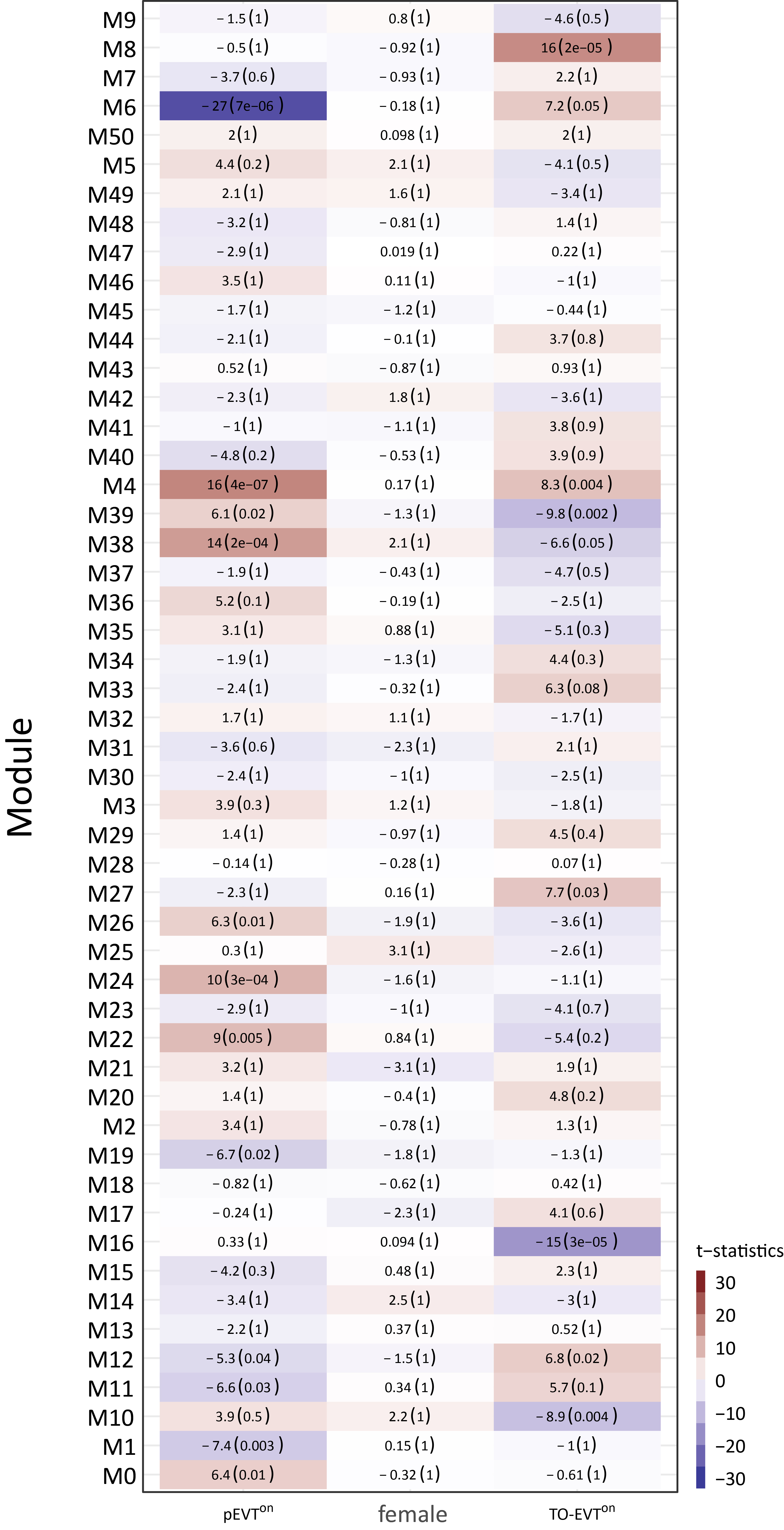
**

**Supplementary Figure 1:** The heatmap presents the association between a module and the three conditions, which are TGFβ activity, fetal sex, and cell origin, from left to right. The t-statistic values displayed indicate upregulation when they are greater than 0 and downregulation when they are below 0, and the corresponding adjusted p-values are shown in brackets. If a module was found to be significantly up- or downregulated in more than one condition, it was excluded from further analysis.

**
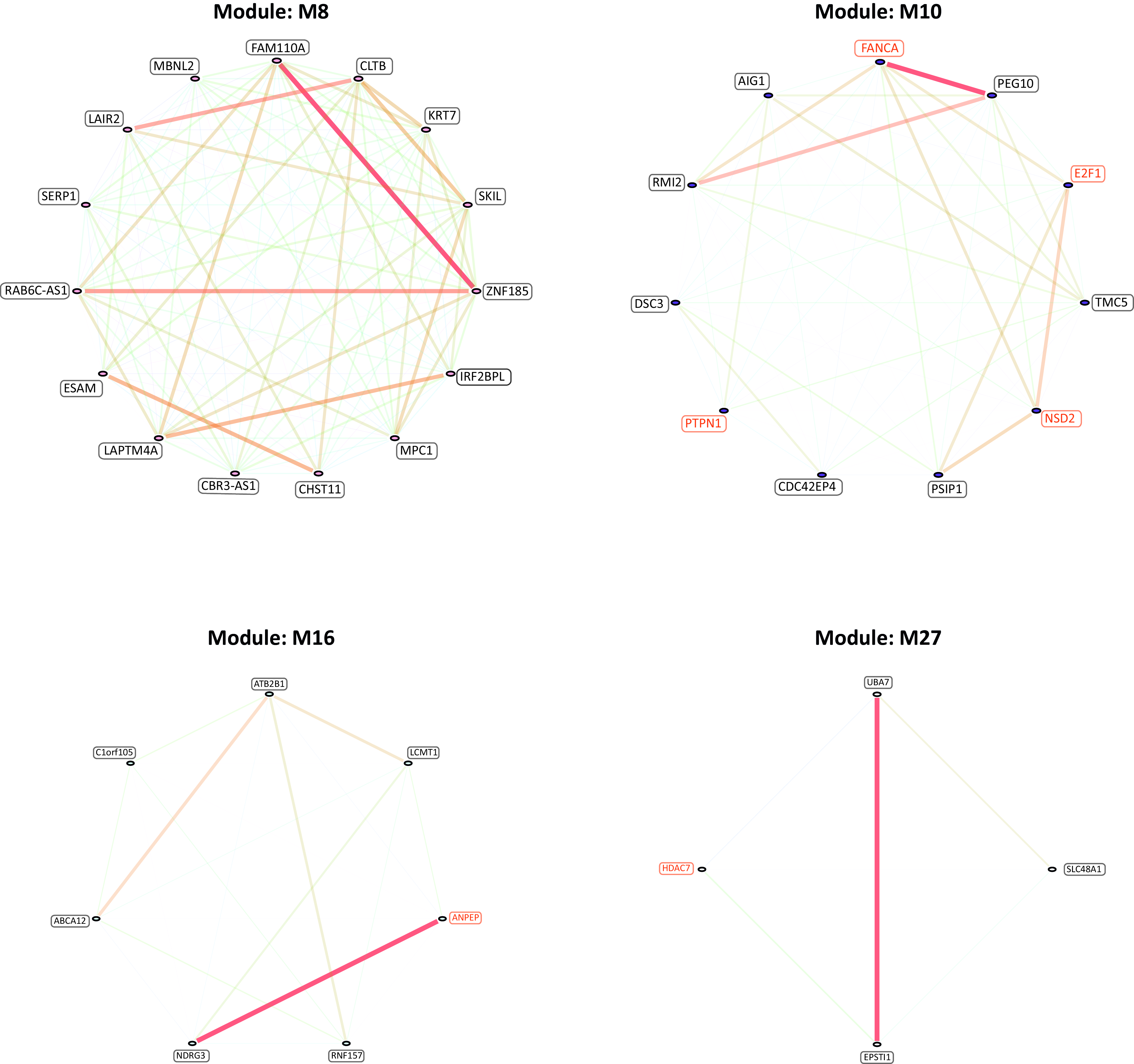
**

**Supplementary Figure 2:** Network of hub genes from TGFβ-associated modules. Hub genes are interconnected by lines that indicate the strength of their interactions. Red labels on the lines signify found interactions in the Drug-Gene Interaction Database analysis.

**
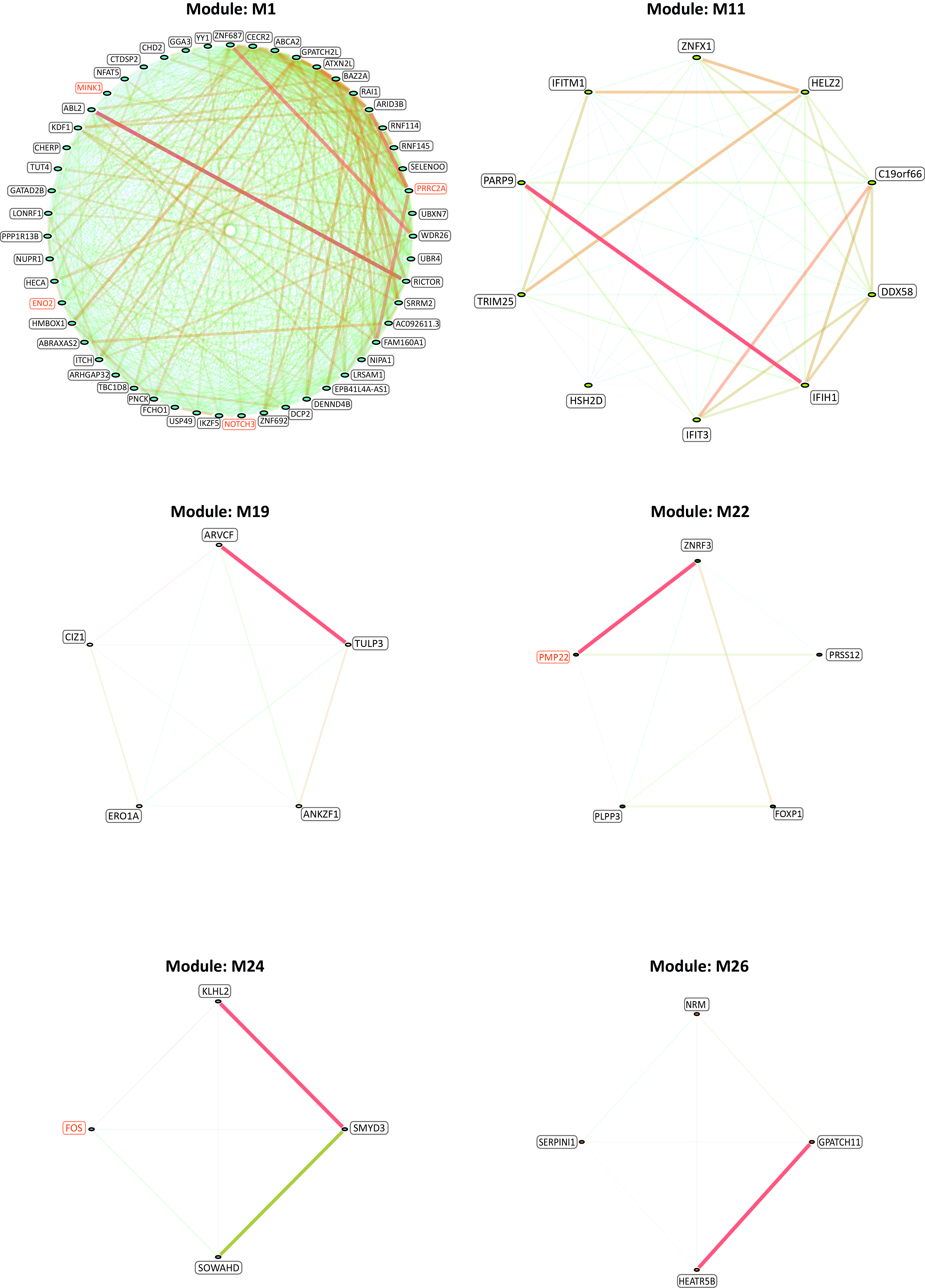
**

**Supplementary Figure 3:** Network of hub genes from cell-origin-associated modules. Hub genes are interconnected by lines that indicate the strength of their interactions. Red labels on the lines signify found interactions in the Drug-Gene Interaction Database analysis.

**
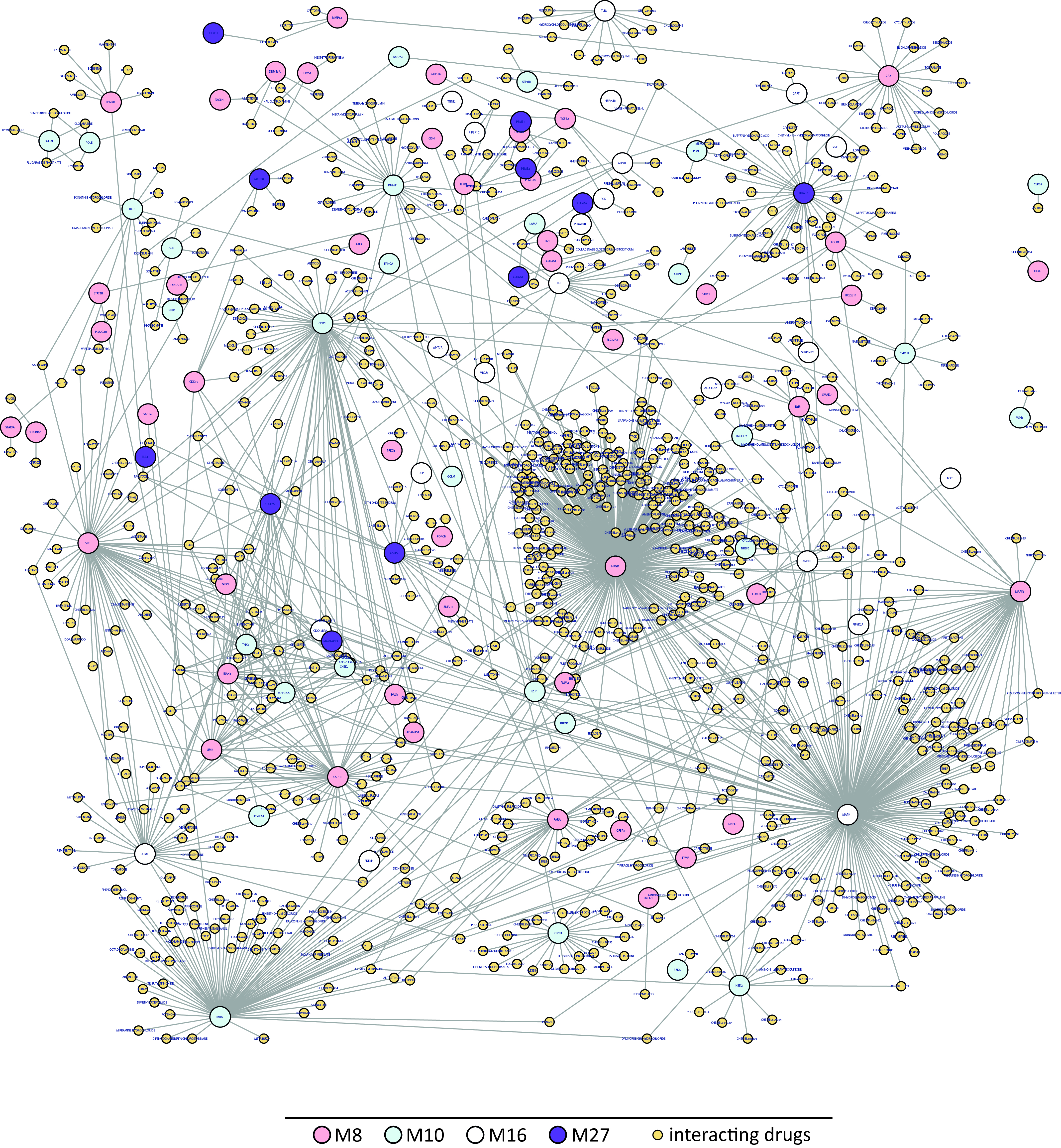
**

**Supplementary Figure 4: Drug-Gene interaction plot for TGFβ-associated modules.** Network plot depicting module network genes and interacting drugs. Module network genes are colored according to the module color. Names of genes and interacting pharmaceuticals are depicted in the plot.
